# Advances and Opportunities in NIR-II Endoscopy: From Diagnosis to Therapeutic Applications

**DOI:** 10.3390/diagnostics16070986

**Published:** 2026-03-25

**Authors:** Jing Luo, Xiaofan Du, Sijia Wang, Cuiping Yao, Jing Wang

**Affiliations:** Institute of Biomedical Photonics and Sensing, Key Laboratory of Biomedical Information Engineering of Ministry of Education, School of Life Science and Technology, Xi’an Jiaotong University, Xi’an 710049, China; 1474642445@stu.xjtu.edu.cn (J.L.); dxf03@xjtu.edu.cn (X.D.); wang_sijia@xjtu.edu.cn (S.W.); zsycp@mail.xjtu.edu.cn (C.Y.)

**Keywords:** second near-infrared (NIR-II) window, endoscopy, minimal invasive surgery, artificial intelligence, telemedicine

## Abstract

Endoscopy refers to the minimally invasive optical visualization and examination of internal structures within the body. Its significance lies in diagnosing intraluminal tissue abnormalities and assisting therapeutics, especially in the gastrointestinal tract. However, conventional optical endoscopes are limited by their insufficient penetration depth. Although endoscopic ultrasound achieves deeper penetration of up to 10 cm, it suffers from compromised spatial resolution. Recent advances have expanded the role of endoscopy from basic mucosal visualization to precision diagnostics, therapeutic assistance, and even intelligent, remote-assisted procedures. An emerging modality, second near-infrared window (NIR-II, 1000–1700 nm) endoscopy, offers deep tissue penetration, reduced scattering, and a high signal-to-noise ratio. This review discusses the clinical requirements of endoscopy across screening, diagnostics and therapeutics. It provides a comparative assessment of current methodologies, and a particular focus is placed on discussing the promising developments in NIR-II endoscopy. Furthermore, we investigate the transformative potential of integrating artificial intelligence and fifth-generation wireless networks into endoscopic practice. The continued evolution and clinical translation of these technologies, particularly NIR-II endoscopy, hold the promise to fundamentally enhance precision medicine in gastroenterology.

## 1. Introduction

**Figure 1:**
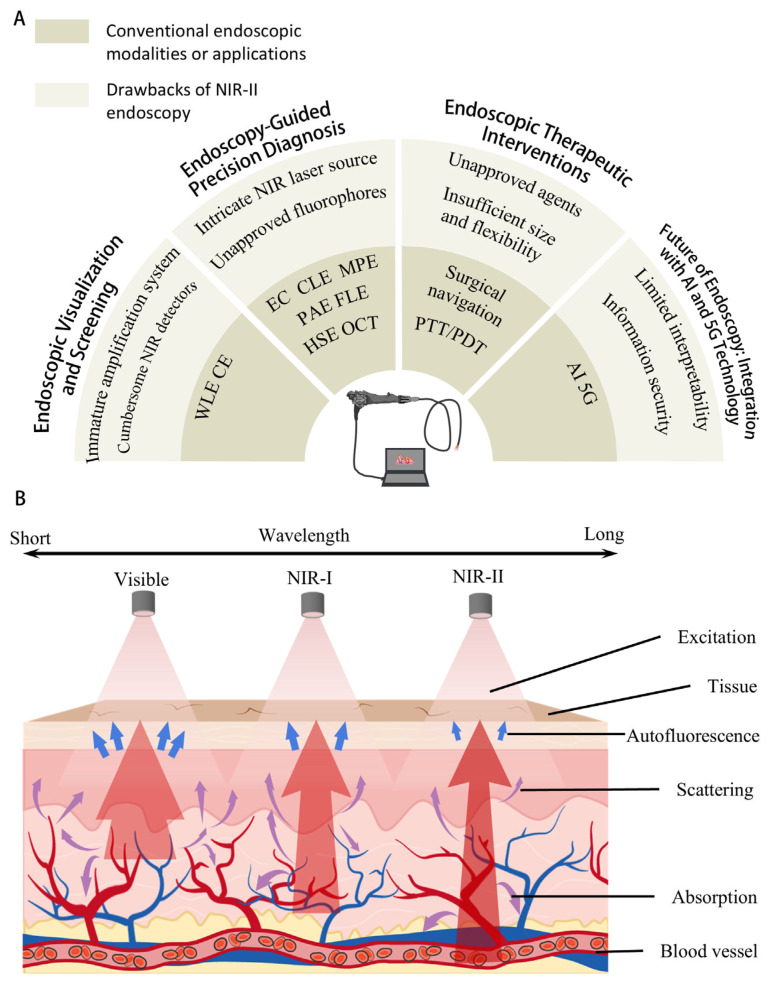
(**A**) Conventional endoscopic modalities and drawbacks of corresponding NIR-II endoscopy. WLE: white-light endoscopy; CE: chromoendoscopy; EC: endocytoscopy; CLE: confocal laser endomicroscopy; MPE: multiphoton endoscopy; PAE: photoacoustic endoscopy; FLE: fluorescence endoscopy; HSE: hyperspectral endoscopy; OCT: endoscopic optical coherence tomography; PTT/PDT: photothermal/photodynamic therapy; AI: artificial intelligence; and 5G: fifth-generation mobile communication technology. (**B**) The mechanism for optical bioimaging. With an increasing wavelength, scattering and spontaneous fluorescence diminish, while the light penetration depth increases, and the image signal-to-noise ratio is enhanced. For the specific tissue penetration depths and imaging performance of each optical window, see [41,42].

Endoscopy is a technique for the optical examination of internal structures. Within medicine, it describes the procedure for inspecting the internal cavities of the human body [1]. It enables gastrointestinal (GI) tract inspection, precancerous lesions detection, surgical operation navigation, and so on, with minimal discomfort for the patient or the need for sedation [2,3,4,5,6,7,8,9,10,11,12]. White-light endoscopy (WLE) is the most prevalent modality in clinical practice, providing essential visualization for initial mucosal screening [9]. Chromoendoscopy and fluorescence endoscopy are also widely applied, offering superior contrast between abnormal and normal tissue. Emerging techniques, including confocal laser endomicroscopy (CLE), endocytoscopy (EC), hyperspectral endoscopy (HSE), multiphoton endoscopy (MPE), endoscopic optical coherence tomography (OCT), and photoacoustic endoscopy (PAE), are being explored in specific clinical domains and demonstrating potential to expand diagnostic capabilities (Figure 1A) [5,11,13,14,15,16,17,18,19]. Conventional endoscopy typically employs wavelengths in the visible range (400−650 nm), where strong tissue absorption, scattering, and autofluorescence limit penetration depth to <3 mm. This results in substantial loss of physiological and pathological information [20,21,22,23].

Expanding the utilizable spectrum of light from the visible region to the near-infrared (NIR) window has greatly facilitated the clinical application of optical technologies [24,25,26]. NIR light exhibits deeper tissue penetration and a higher signal-to-noise ratio (SNR) compared to visible-wavelength light, owing to decreased photon absorbance, scattering, and autofluorescence. Of course, these advantages are dependent on tissue type, optical properties, and other specific factors [21,22,23,27]. The first near-infrared window (NIR-I, 700–900 nm) has been applied in clinical endoscopic examination and shown greater clinical diagnostic and therapeutic efficacy [28,29]. In 2009, Dai et al. revealed a new bioimaging sub-window, commonly known as the second near-infrared window (NIR-II, 1000–1700 nm) [30,31]. Although water absorption in the NIR-II window is relatively stronger at specific wavelengths (e.g., 970, 1200, 1450 nm), drastically reduced tissue scattering and autofluorescence play dominant roles (Figure 1B) [20,27,32]. Numerous innovative fluorophores and imaging techniques in the NIR-II have been developed [30,33,34,35,36,37,38,39,40]. However, little research has focused on their application in endoscopy.

Benefiting from the ultralow background signal characteristic of the NIR-II window (nearly zero beyond 1500 nm), NIR-II endoscopy permits ultrahigh-sensitivity detection beyond the capability of traditional endoscopy [20]. Meanwhile, compared to other bioimaging applications, NIR-II endoscopy is subject to unique constraints and practical requirements, including real-time visualization, compatibility with endoscopic optical hardware, and high spatial resolution at clinically relevant depths [21,22]. These are critical for meeting medical demands in three key clinical fields: endoscopic screening, diagnosis, and therapeutics [43]. Building on this context, this review first outlines the current status, strengths, and limitations across these three domains of conventional endoscopy, providing a foundation for the development and advancement of NIR-II endoscopic technology. Furthermore, the integration of two emerging technologies, artificial intelligence (AI) and fifth-generation mobile communication technology (5G), is also discussed. Their potential to equip endoscopes with intelligent and remote functions unlocks new possibilities for NIR-II endoscopy. We aim to suggest future directions for research on NIR-II endoscopy and facilitate its translation into clinical practice.

## 2. Methods

A systematic literature search was conducted using Google Scholar to identify relevant studies published between January 2015 and December 2025. The search strategy employed a combination of keywords related to both imaging technology and endoscopic modalities, including: “NIR-II”, “Second Near-Infrared”, “Near-Infrared II”, “Near Infrared II”, “endoscopy”, “endoscopic”, “endoscopically”, “endoscope”, “Endomicroscopy”, “Ureteroscopy”, “Cystoscopy”, “Colposcopy”, “Hysteroscopy”, “Rhinolaryngoscopy”, “Bronchoscopy”, “Endoscopic computer-assisted diagnosis”, “high-resolution endoscopy”, “ultra high-resolution endoscopy”, and “magnification endoscopy”.

This review focused on original studies investigating the application of NIR-II imaging in endoscopic techniques, particularly those reporting on imaging performance, technological advancements, diagnostic accuracy, or clinical feasibility. Priority was given to peer-reviewed original research articles, clinical trials, and prospective or retrospective studies with clear relevance to the integration of NIR-II imaging with endoscopic systems.

Studies were included if they specifically addressed the use of NIR-II fluorescence imaging in combination with any form of endoscopy and provided original data on methodology, imaging outcomes, or clinical applications. Articles not officially published, conference abstracts, editorials and studies lacking direct relevance to NIR-II endoscopic imaging were excluded. A descriptive synthesis of the evidence was performed, highlighting the principal advancements and potential directions for future research in the convergence of NIR-II imaging and endoscopic technologies.

## 3. Endoscopic Visualization and Screening

### 3.1. Conventional Endoscopy

**Figure 2:**
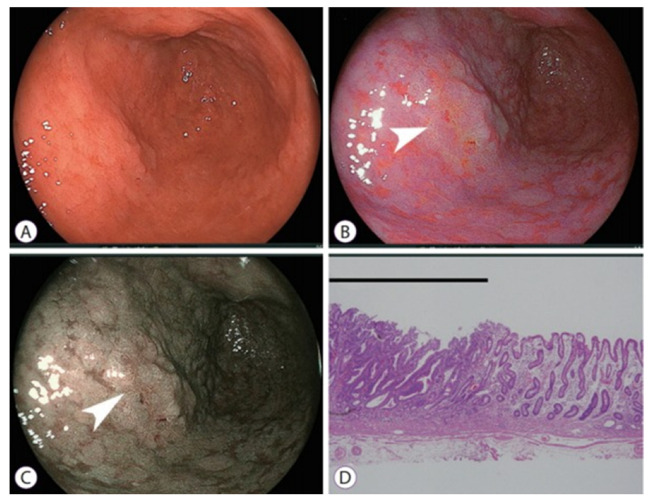
Early gastric cancer in the antrum, where the intestinal metaplasia spreads over broad areas. (**A**) White-light imaging does not demonstrate findings suggestive of malignancy. (**B**) The linked color imaging shows an orange lesion (arrow), suggestive of cancer, surrounded by purple mucosa. (**C**) The blue laser imaging shows a brown lesion (arrow) surrounded by green mucosa. (**D**) Pathological examination of the resected specimen shows well-differentiated adenocarcinoma (black line) adjacent to the intestinal metaplasia (hematoxylin and eosin, ×40). Reprinted from ref. [45], licensed under CC BY-NC 3.0 (http://creativecommons.org/licenses/by-nc/3.0 accessed on 11 January 2026). Copyright © 2018 Korean Society of Gastrointestinal Endoscopy.

The primary purpose of endoscopy is to visualize anatomical structures and perform basic screening [43]. In clinical practice, endoscopic screening aims to achieve timely and accurate lesion detection and precise delineation of abnormal tissues, all under the constraints of real-time imaging and procedural efficiency. WLE and chromoendoscopy are two primary techniques used for this purpose. With their large field of view (FOV) and shallow penetration depth, these techniques are ideally suited for superficial mucosal examination of luminal organs [9,16]. The key distinction between the two modalities lies in contrast enhancement: WLE uses unmodified white light (typically an LED, Halogen or Xenon-based light source), whereas chromoendoscopy employs exogenous dyes (dye-based chromoendoscopy) or modified light spectrum (virtual chromoendoscopy, VCE) to highlight mucosal abnormalities (Figure 2) [1,4,14,16,44]. Clinically available dyes and VCE systems are listed in Table 1 and Table 2, respectively.

To improve the performance of clinical endoscopic screening, particularly in terms of lesion detection, tissue delineation, and real-time imaging reliability, key optical and hardware parameters play critical roles in determining diagnostic accuracy [72,73,74,75,76,77]. With the advancement of camera sensors, the definition of endoscopic imaging systems has evolved from standard definition (SD, 640 × 480 pixels) to high definition (HD, 1280 × 720 or 1366 × 768 pixels), Full HD (1920 × 1080 pixels), and even Ultra HD (UHD or 4K, 3840 × 2160 pixels) [1,78]. Furthermore, the whole imaging chain, including optics, monitors, image documentation modes, and video or static images, is important as well [1]. Clinically, dye-based chromoendoscopy is a relatively “old” but cheaper method [73,79,80]. It remains superior in certain applications, such as the depth assessment of colorectal cancer and the detection of dysplasia in inflammatory bowel disease (IBD) [81,82,83,84]. By comparison, WLE and VCE offer distinct strengths as initial, complementary screening tools for efficient lesion identification and localization, owing to their clinical simplicity and real-time usability. These preliminary findings can then be validated and further characterized using dye-based chromoendoscopy or other targeted endoscopic procedures [85].

### 3.2. NIR-II Endoscopy

Leveraging the NIR-II window’s ultralow background signal and deeper tissue penetration, NIR-II endoscopy can achieve high-resolution imaging at depths beyond the reach of traditional endoscopy. However, the majority of clinically available endoscopes are currently optimized predominantly for high-resolution visible light imaging, with limited transmittance of NIR light [21,22]. Most of the existing amplification and focusing optical systems for NIR-II endoscopy are fixed-focus and only support low-level magnification (range from ×2.5 to ×25), significantly inferior to commercially available magnifying endoscopes [27,75,86,87,88]. To address these challenges, the design of a high-level magnification and zooming endoscopic system needs to consider the unique optical properties of NIR light, such as chromatic aberration, transmittance and photothermal properties. Chen et al. designed a wide-spectrum zoom optical system using ZEMAX. The system, with a zoom range of 25 mm to 150 mm and a working spectral band of 400 nm to 1700 nm, achieves a full-field MTF ≥ 0.3 at a spatial frequency of 100 lp/mm. Although it has not yet been clinically verified, it demonstrates promising potential for application in NIR-II endoscopy [89].

The acquisition of physiological information via endoscopy is heavily dependent on image quality. In the NIR-II window, imaging typically requires indium gallium arsenide (InGaAs) cameras, which are costly and susceptible to dark currents [90]. Currently, commercially available InGaAs cameras offer imaging capabilities comparable to silicon detectors, and numerous methods have been developed to overcome their inherent barriers [91]. Sun et al. demonstrated that reducing the intrinsic layer thickness of InGaAs detectors within a specific range can minimize dark currents to the nanoampere (nA) level [92]. Wu et al. noted surface passivation reduces leakage and noise [93]. Furthermore, AI and heteroepitaxy techniques prove promising [94,95]. Emerging techniques, such as two-dimensional (2D) and organic photodetectors, are potential alternatives to InGaAs detectors [96,97,98,99,100].

Another revelation for NIR-II endoscopy is utilizing the selective tissue absorption of specific NIR-II wavelengths to expand the application of VCE. RDI (red dichromatic imaging) is a kind of novel VCE mode included in the EVIS X1 system (Olympus). Compared to traditional NBI (narrow-band imaging, centered at 415 nm and 540 nm), red (620–640 nm) and amber (595–610 nm) light, with a longer wavelength, is used to reach deep into the mucosa at approximately 1.0–1.5 mm and display deep blood vessels and mucosa at a constant brightness in RDI mode [64,66]. This performance is particularly valuable for endoscopic screening of superficial and submucosal lesions in the GI tract, such as esophageal varices and ulcerative colitis, as well as for hemostasis of acute GI bleeding [67]. Its advantages can be further expanded by NIR-II light. Additionally, tissue possesses unique absorption and scattering characteristics in the NIR-II window. For example, lipids exhibit higher contrast at 1150 nm and 1210 nm [101]. This characteristic holds significant clinical potential for the screening and characterization of atherosclerotic plaques in esophagus-adjacent vascular structures and lipid-rich neoplastic lesions in the GI tract.

## 4. Endoscopy-Guided Precision Diagnosis

While WLE and chromoendoscopy enable preliminary morphological assessment, a further requirement exists for in vivo histology to resolve microstructural features, and for molecular profiling to enable accurate diagnosis and staging. In parallel, precise depth-resolved imaging of lesions is essential for guiding subsequent therapeutic interventions. To address these distinct diagnostic objectives, various emerging endoscopic modalities are being developed, each with unique technical requirements and validation standards.

### 4.1. Conventional Endoscopy

#### 4.1.1. In Vivo Histological Assessment

CLE and EC are two techniques renowned for their cell-level imaging capabilities. These technologies enable real-time micron-scale imaging and support in vivo “optical biopsy”, providing a promising alternative to traditional ex vivo histology. Both have been approved for clinical use by the U.S. FDA [102,103].

**Figure 3:**
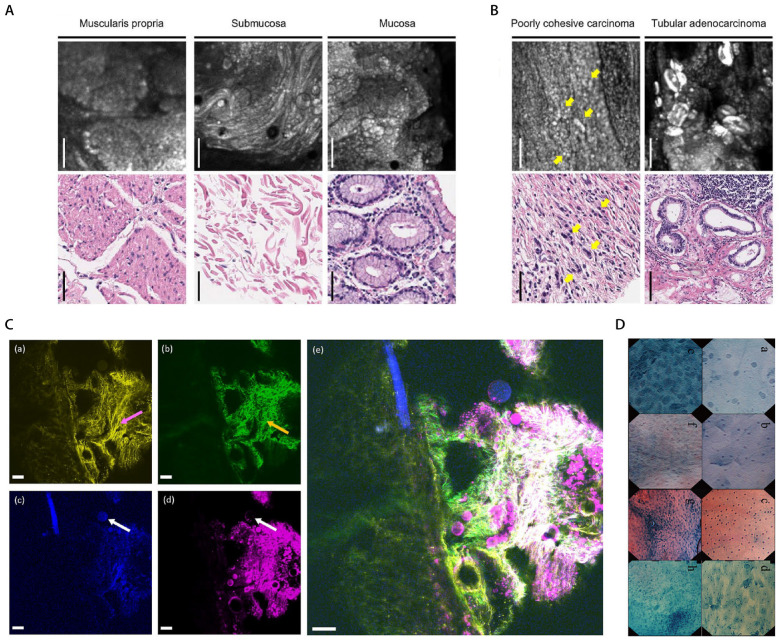
(**A**) Representative confocal laser endomicroscopy system (CLES) images alongside corresponding hematoxylin and eosin (H&E) images of non-neoplastic gastric tissue regions. Scale bar, 50 μm. CLES images obtained from non-neoplastic gastric tissue clearly demonstrated the characteristics of the mucosa, submucosa, and muscularis propria, respectively. (**B**) Representative CLES images and H&E images corresponding to regions of gastric cancer tissue. Yellow arrow, tumor cells. Scale bar, 100 μm. Tubular adenocarcinoma appeared as bright round or ovoid structures on CLES images. In contrast, poorly cohesive carcinoma presented as scattered small bright dots on CLES images, consistent with its H&E histomorphology. (**A**,**B**) are adapted from ref. [111], licensed under CC BY 4.0 (http://creativecommons.org/licenses/by/4.0/ accessed on 15 January 2026). Copyright © 2024 Seokhwi Kim et al. (**C**) 2-photon fluorescence (TPF)/second harmonic generation (SHG)/3-photon excited fluorescence (3PEF)/third harmonic generation (THG) images of ex vivo human gastric tissue. (**a**) TPF image showing elastic fibers (pink arrows); (**b**) SHG image showing collagen fibers (yellow arrows); (**c**) 3PEF image and (**d**) THG image showing adipocytes (white arrows); (**e**) Merged image of TPEF/SHG/3PEF/THG. Scale bar: 100 μm. Reprinted from ref. [112]. Copyright © 2022 Chinese Physical Society. (**D**) Representative endocytoscopy images. (**a**–**d**) Normal squamous epithelial cells. (**e**–**h**) Esophageal squamous cell carcinoma. The instruments and corresponding magnifications are XEC120U (×1125), XEC300F (×450), XGIF-Q260EC1 (×450), and GIF-Y0002 (×380), respectively. All specimens were stained with methylene blue. Lower-power endocytoscopy system of the cancerous region demonstrates significantly higher cell density than that of normal squamous epithelium. Using the XEC120U system, irregular cell distribution and marked cellular heterogeneity are observed, with nuclei varying in staining intensity, size, and shape. Reprinted with permission from ref. [113]. Copyright © 2010 Youichi Kumagai et al.; Japan Gastroenterology Endoscopy Society.

CLE enables in vivo histological assessment with up to ×1000 magnification (Figure 3A,B) [104]. The systems are categorized into endoscope-based (eCLE) and probe-based (pCLE) systems, of which pCLE is a commercially available system [105]. MPE shares similarities with CLE. Signal generation scales with the square of the excitation irradiance, confining photon emission to the focal point (Figure 3C). This reduces the risk of tissue damage, photobleaching and fluorescence emission outside the focal plane. However, MPE imaging quality is limited by the laser source, which requires extremely short pulses (<100 fs) and higher peak power [106,107,108,109,110].

EC (Olympus, Tokyo, Japan) is a contact-based imaging technique that enables the observation of subcellular components, including nuclei and cytoplasm (Figure 3D). The fourth-generation EC, the GIF-H290EC gastroscope, integrates magnifying NBI and supports endocyto observation at 520× continuous zoom magnification, with an observation range of 570 μm × 500 μm and an outer diameter of 9.7 mm [114].

Collectively, CLE and EC exhibit substantial clinical value for real-time in vivo microstructural imaging, with key applications including the evaluation of epithelial barrier function and vascular permeability, the assessment of mucosal healing in IBD, and the early diagnosis of GI tract neoplasms [102,115,116]. Compared with conventional endoscopic approaches reliant on ex vivo biopsy for definitive diagnosis, these techniques optimize diagnostic decision-making by providing real-time in vivo histology-like imaging and streamline clinical workflows through on-procedure real-time diagnosis, eliminating the need for delayed pathological confirmation.

#### 4.1.2. Functional and Molecular Imaging

Fluorescence endoscopy is a typical functional/molecular contrast imaging modality, which generates specific contrast based on endogenous tissue fluorophores or exogenous molecular fluorescent probes to reflect the functional and molecular characteristics of lesions, thus realizing targeted identification of abnormal tissues [117,118]. Clinically used endogenous fluorophores include nicotinamide adenine dinucleotide (phosphate), flavin adenine dinucleotide, and collagen, whereas exogenous fluorescence systems primarily rely on NIR imaging with indocyanine green (NIR/ICG). HSE is another molecular imaging modality. It integrates spectroscopy and imaging, reflecting physiological, compositional, and morphological information for diagnosis [119].

Compared to fluorescence endoscopy, HSE is less frequently used in clinical settings. Its main limitations include cladding artifacts and motion-induced blurring during data acquisition [120,121]. These issues can potentially be mitigated through advanced computational tools [122,123,124,125]. Both modalities hold significant clinical value for tasks such as early neoplastic lesion detection, invasion depth assessment and intraoperative navigation [5,126,127,128,129,130,131,132,133]. Compared with morphological endoscopy, they improve diagnostic accuracy via functional/molecular tissue characterization, enabling real-time intraprocedural evaluation and reducing unnecessary biopsies.

#### 4.1.3. Depth-Resolved Imaging

**Figure 4:**
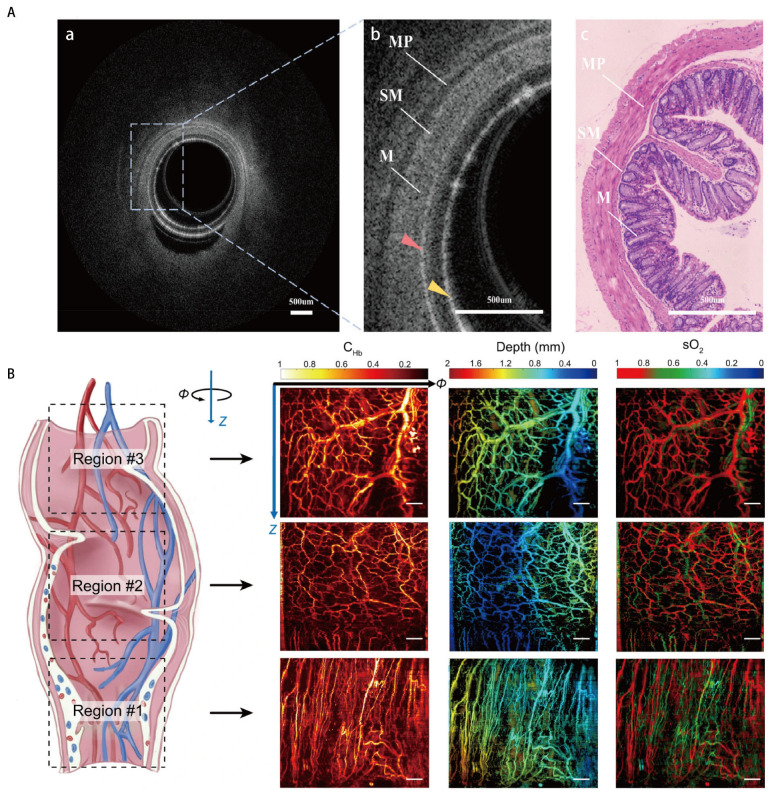
(**A**) Endoscopic optical coherence tomography (OCT) images of the mouse rectum and a comparison with histopathological images. (**a**) a transverse cross-sectional OCT image. (**b**) An enlarged view of the dashed box in (**a**) M: mucosa; MP: muscularis propria; and SM: submucosa. (**c**) A histopathological image of the same mouse rectum. The outer and inner walls of the sheath are indicated by red and yellow arrows, respectively. Texture of the mouse rectum presented in the magnified OCT image is validated in a representative histopathological section. Reprinted with permission from ref. [139]. Copyright © 2023 Wiley-VCH GmbH. (**B**) In vivo optical focusing photoacoustic endoscopy imaging results on the hemoglobin concentration (C_Hb_), depth, and oxygen saturation (sO_2_) of a rat rectum in three different regions. Region #1 is near the anus, and regions #2 and #3 are located 2 cm and 3 cm deep from the anus, respectively. The imaging results exhibit significantly different vascular network profiles. Scale bar: 1 mm. Reprinted with permission from ref. [146], licensed under CC BY 4.0 (https://creativecommons.org/licenses/by/4.0/ accessed on 15 January 2026). Copyright © 2022 Yizhi Liang et al.

Endoscopic OCT and PAE empower endoscopy with the ability for depth-resolved imaging. Endoscopic OCT enables cross-sectional subsurface imaging of tubular organs and cavities (Figure 4A) [134,135,136,137]. The lateral resolution of endoscopic OCT is determined by focusing optics, while the axial resolution depends on the light source [138]. A ~1300 nm swept-source with a ~100 nm full-width at half-maximum (FWHM) bandwidth is commonly used [139,140,141,142,143]. PAE is based on the photoacoustic (PA) effect (Figure 4B) [144,145]. The lateral resolution of PAE is determined by either optical focusing (optical-resolution PAE) or acoustic detection (acoustic-resolution PAE), and the axial resolution is determined by the bandwidth of the ultrasound detector [146,147,148].

Both modalities possess the ability for three-dimensional (3D) spatial imaging [149,150]. Leveraging this ability, these technologies are tailored for specific clinical tasks. For instance, endoscopic OCT provides 3D virtual hematoxylin- and eosin (H&E)-stained images, directly informing surgical resection decisions [134]. Meanwhile, PAE enables functional imaging of capillary blood oxygen saturation, guiding biopsy targeting and monitoring anti-angiogenic therapy responses [146].

Simple fluorescence imaging is a wide-field imaging method that does not require scanning and is sometimes combined with other endoscopy modalities in specific clinical contexts. In contrast, advanced endomicroscopy technologies, including HSE, CLE, OCT, PAE, and MPE, often operate with a narrow FOV. They rely on focused laser light to scan superficial tissue layers in a certain pattern [1,151,152]. This comes with hurdles, including prolonged imaging time and requirements for specialized operator training [115,121,137,141,153,154,155,156,157]. Currently, systems for advanced endomicroscopy are expensive and not routinely used in clinical practice [1]. Table 3 summarizes the comparative performance of these modalities.

### 4.2. NIR-II Endoscopy

Much research has been conducted to combine the NIR-II window with fluorescence endoscopy and other endomicroscopy techniques. To date, all these studies remain at the preclinical or animal experimental stage. Nevertheless, the results demonstrate the significant advantages of the NIR-II window. In 2019, Cheng et al. pioneered NIR-II endoscopic imaging using a dichroic endoscope capable of simultaneous visible- and NIR-II-image acquisition, with millimeter-scale field adjustment (minimum 4 mm^2^) and ~20 μm resolution. Their system successfully delineated rat orthotopic colorectal tumors using ICG-bevacizumab conjugate [167]. Other attempts have been made to apply the NIR-II window to CLE. Qian et al. developed a confocal NIR-II fluorescence microscope, enabling the reconstruction of a 3D volume of cortical vasculature at depths of up to ~500 μm, with the ability to resolve capillary vessels less than 7 μm in diameter [27]. Dai et al. achieved 3D NIR-II molecular imaging in a mouse ovary model, demonstrating a penetration depth of 900 µm and micrometer-level spatial resolution (18 µm “z” interval between layers) [88]. For MPE, third-order nonlinear processes, including three-photon excitation fluorescence (3PEF), third harmonic generation (THG), and coherent anti-Stokes Raman scattering (CARS), tend to require NIR-II excitation wavelengths [106,107]. Kudlinski et al. showed a multimodal nonlinear micro-endoscope with a 2 mm diameter. Compared to two-photon fluorescence (TPF), 3PEF images exhibit enhanced spatial resolution and edge sharpness due to the higher power density (Figure 3C) [161]. However, the limitations of femtosecond pulsed lasers are stronger for third-order nonlinear processes [161,168]. Additionally, the NIR-II window has also shown great potential in endoscopic depth-resolved imaging. Jiang et al. examined a remarkably compact endoscope, which integrates photoacoustic imaging (PAI), OCT, and ultrasound (US). For OCT, a broadband light source with a center wavelength of 1310 nm (75 nm FWHM) was employed. During in vivo imaging of a mouse ear, PAI and OCT successfully visualized the microvasculature and intricate perivascular structures, respectively. PAI mapped blood vessels with the highest contrast, and OCT identified the epidermis, dermis, and cartilage [101].

Looking at these studies as a whole, there are distinct limitations to integrating the NIR-II window with specific endoscopic modalities. A primary challenge for NIR-II endoscopy lies in fluorescent probes. The dyes employed are either clinically approved NIR-I agents (e.g., ICG, IRDye800CW) that emit weakly in the NIR-II range, or novel probes developed in-house [169,170]. Most preclinical studies prioritize technological performance (e.g., ultra-high quantum efficiency) (Table 4), yet clinical translation requires a comprehensive assessment of safety, practicality, and efficacy [21,171,172,173,174]. No novel tumor-targeted NIR-II probes or photosensitizers have entered clinical trials. Beyond technical and biocompatibility concerns, regulatory approval barriers are a bottleneck for translating NIR-II agents to the clinic. The FDA and EMA enforce stringent requirements for molecular imaging agents, mandating rigorous preclinical and clinical testing to confirm biocompatibility, consistent performance, and favorable risk-benefit profiles [175,176]. These requirements are compounded by the lack of standardized evaluation criteria and dedicated regulatory guidance for NIR-II endoscopic applications. Certainly, clinical exploration of NIR-II endoscopy can be supported by utilizing approved NIR-I agents temporarily, including ICG, IRDye800CW, and methylene blue.

On the hardware side, NIR high-repetition-rate pulsed lasers and optimized scanning methods are essential for speed, quality, and artifact reduction, directly impacting clinical practicability [101,161,168,177,178]. To address these challenges, incremental innovation is recommended: NIR-II imaging can be integrated as an add-on channel to upgrade existing multispectral systems, explore novel spectral applications, and merge modalities to capitalize on complementary strengths [174]. While current NIR-II cameras are costly, costs will decrease with standardization, mirroring the trajectory of NIR-I platforms [21].

**Table 4:** Some research focusing on NIR-II fluorophores.

Type	Name	Excitation/Emission (nm)	QYs	Hydrophilic Modification	Applications	Targeted Elements
Cynaine dyes	IR-32p [179]	1020, 1064/1120	/	Coupled polyethylene glycol.	In vivo targeted brain glioma imaging.	cRGDfK
	BIT NPs [180]	1012/1120	0.42%; (IR-1061 = 1.7%)	Integrating bovine serum albumin.	Spatiotemporally specific diagnosis and combination therapy of tumors.	Nonspecific
	IR-TPP-1100 [181]	1020/1100	0.83%; (IR-1061 = 1.7%)	Encapsulated with F-127.	NIR-II FL/NIR-II PA imaging-guided PTT/PDT.	TPP
	ICG-Herceptide [182]	Similar to free ICG	/	/	In vivo tumor imaging and image-guided surgery.	Herceptide peptide
	AIR-PE [183]	808/1080 ± 10	0.27% (in water)	The mixture of PLGA and Eudragit S100. Coating.	Real-time NIR-II imaging of IBD.	Nonspecific
	LZ-1105@Ham [184]	1064/1105	/	Human non-small cell lung cancer cell membrane coating.	NIR-II FL/PA/PT imaging and PTT.	Cell lung cancer cell membrane
	CyN-Ome [185]	1064/	0.1%; (IR786 = 19%)?	Encapsulated with liposome.	PTT for tumor ablation.	Nonspecific
	NIRG-2 [186]	850/940	/	Introducing the hydrophilic sulfonic group.	Visualizing the tumor’s lymphatic metastasis and precise tumor resection.	G-quadruplex (G4)
D-A dyes	TPGS-NT-4 NPs [187]	808/1050	3.46%	d-α-tocopheryl polyethylene glycol succinate coating.	Angiography and localized photothermal therapy.	Nonspecific
	TQ-100 [188]	808/1006	0.1%; (IR-26 = 0.5%)	Coupled with protein.	Tumor therapy and neuromodulation.	RGD
	OBADC-TPA [189]	660, 685, 808/900–1100	/	Amphiphilic polymers coating.	PA/NIR-II FL imaging-guided PTT/PDT of lymphoma.	9-NH2-SA
	B-ToMeT NCs [190]	/970	9.7% (in crystal state); 28.2% (in water)	/	Real-time monitoring of intestinal vessels.	Nonspecific
	TTX-P [191]	808/920	/	/	NIR-II FL imaging of diabetic liver injury.	Phosphate group
QDs	CD-AuNCs [192]	808/above 1000	0.098% (in water); (IR-26 = 0.05%)	β-cyclodextrin coating.	Profiling of early-stage acute kidney injury.	Nonspecific
	DPTPzIr NPs [193]	808/1108	0.15%	DSPE-mPEG2000 coating.	NIR-II FL/NIR-II PA/NIR-II PT imaging-guided NIR-II PTT/PDT.	Nonspecific
	Syn-Ag2S NC [194]	808/1220	46 ± 2%	Synchronous passivation with MgCl2.	Deep lymph node imaging.	Nonspecific
	Nd@Y-FA NPs, Er@Y-PEG NPs [195,196]	808/1060, 1525	/	The mixture of mPEG-NH2 and 8 Arm-PEG-NH2 coating.	Determining the metastatic status of sentinel lymph nodes.	FA (targeting folate receptors)
Else	PPy-TAT-R848-HA NC [197]	808, 1064/	/	Hyaluronic acid coating.	Induced tumor ablation; activated ICD and immunotherapeutic agents.	TAT peptide
	BSA@TT NPs [198]	760, 808/960	3.82%; (ICG = 1.7%)	Bovine serum albumin coating.	Microvascular visualization and tissue discrimination.	Bovine serum albumin
	5SGNPs NPs [199]	/above 1200	/	Constructing amphiphilic lipid nanocarrier.	Accurate thrombus visualization and PTT.	Bis–serotonin (bis-5HT)
	BTC12 NPs [200]	808, 1064/940	/	Assembled with 1,2-dimyristoyl-sn-glycero-3-phosphocholine.	NIR-II FL/NIR-II PA imaging-guided NIR-II PTT.	Nonspecific
	BM dyes [201]	808/870–930	ΦF = 10.4–18.0% in DCM (dichloromethane)	/	Cerebral vasculature and lymphatic vessels imaging; detecting subtle cerebral capillary damage.	Nonspecific
	MYM [202]	808/extended to 1100	/	Encapsulated in exosomes derived from 293F cells.	Diagnosis and therapeutic treatment of glioblastoma.	iRGD peptide
	DK@RA-PEG [203]	808/~917	0.11%	Conjugated to the polymer N3-PEG2000-NHS.	NIR-II FL imaging guided PDT for rabies.	Aptamer OF RABV glycoprotein
	P-INT [204]	/	/	DSPE-PEG2000-COOH coating.	Neuroimaging and tumor imaging of OCT.	Nonspecific
	MSINPs [205]	1064/	/	Encapsulated with a macrophage membrane.	PA molecular imaging of neuroinflammation.	Macrophage membrane
	BIS-NPs [206]	808/902	2.86%	Coassembled with amphiphilic polymers.	NIR-II FL imaging and PDT of colon cancer.	Nonspecific
	DNHFD [207]	808/1071	/	/	NIR-II FL imaging and loading anticancer drug.	INF-γ aptamers
	DFFP [208]	808, 980/1050, 1550	/	Loading elements onto hydrophilic DCNP.	Real-time evaluation of the Fenton reactivity.	Fe^2+^
	64Cu-NODAGA-uFSH-CH1055 [209]	808/	/	/	PET/CT and NIR-II imaging of various tumors.	Urofollitropin (uFSH)

FL: fluorescence, PA: photoacoustic, PTT/PDT: photothermal/photodynamic therapy, PT: photothermal, IBD: inflammatory bowel disease, ICD: immunogenic cell death, OCT: optical coherence tomography, PET/CT: positron emission tomography/computed tomography. Unless otherwise noted, all works listed above are yet to be clinically validated.

## 5. Endoscopic Therapeutic Interventions

Therapeutics represents another critical application of endoscopic imaging. While conventional flexible endoscopes are widely used in the GI tract for perforation repair, foreign body removal, drug delivery, and premalignant polyp resection, this section focuses specifically on image-guided therapeutic strategies [43]. In particular, we highlight two clinically relevant directions: intraoperative surgical guidance and light-activated therapies, including photothermal therapy (PTT) and photodynamic therapy (PDT).

### 5.1. Surgical Guidance

**Figure 5:**
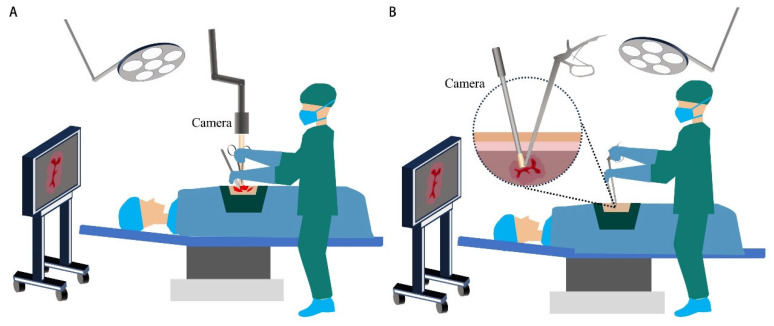
Schematic illustration of clinical surgery. (**A**) Open surgery. (**B**) Laparoscopic surgery. Laparoscopic devices integrate imaging and other surgical instruments into a single system, reducing surgical trauma and facilitating patient recovery.

Mühe’s first laparoscopic cholecystectomy in 1985 marked the advent of minimally invasive surgery (MIS) [210]. This technique aims to minimize incision size and number, thereby reducing soft tissue damage and accelerating patient recovery (Figure 5) [12,211]. MIS encompasses both robotic and nonrobotic approaches [7]. Nonrobotic surgery is often termed laparoscopic, thoracoscopic, or keyhole surgery. Surgeons insert an endoscope equipped with visualization and operation tools through small incisions or natural orifices [212]. In robotic surgery, surgeons operate via a console that controls robotic arms. The incisions, known as ports, typically range in size from 3 mm to 12 mm [213].

To our knowledge, fully endoscope-integrated NIR-II systems for MIS remain unavailable. At present, only open-field NIR-II imaging and ex vivo back-table NIR-II imaging platforms have been evaluated in human subjects [21]. Tian et al. developed an optical imaging instrument integrating a visible multispectral imaging system with NIR-II and NIR-I fluorescence detection (using ICG) to guide the surgical resection of primary and metastatic liver tumors in 23 patients. They demonstrated that intraoperative NIR-II imaging, compared to the NIR-I, achieved higher tumor-detection sensitivity (100% vs. 90.6%), a greater tumor-to-normal-liver-tissue signal ratio (5.33 vs. 1.45), and an improved tumor-detection rate (56.41% vs. 46.15%) [214]. Zhu et al. developed a nanosystem based on NIR-II aggregation-induced emission (AIE) molecules for synergistic fluorescence and chemiluminescence imaging. This approach guided the surgical resection and precise elimination of tumor foci. The designed AIE molecule exhibited stable fluorescence with a high quantum yield of up to 3.95% [215]. Furthermore, several studies about NIR-II imaging surgical navigation have consistently produced favorable outcomes, including cancer localization, surgical margin assessment, and so on [25,216,217].

The diameter and length depend on the intended use of the endoscopes [1]. Due to the limited space in various areas for MIS, the flexibility and size of surgical instruments are important. Additionally, 3D visualization has been used in laparoscopic and robotic surgeries where haptic feedback and depth perception are limited and has become a component of European clinical roadmaps [1,218,219,220,221]. The most renowned da Vinci surgical robotic platform features stereo vision of the surgical field at the master console. Surgeons can interact with anatomy using 7-degree-of-freedom (DOF) devices called EndoWrist [222]. Kim et al. developed a wireless controller to simultaneously operate a proposed endoscope system comprising a 4-DOF ECS and a compact 3D endoscope. The images provide clear stereo vision, enabling 3D visualization during surgery [223]. For NIR-II endoscopes, miniaturization and flexibility of NIR readout and laser devices can make them more suitable for MIS. Moreover, manufacturing and assembly technologies, mechanism types, biomaterials, actuation principles, standards, and regulations all influence the performance of NIR devices, necessitating further research [7,156]. Three-dimensional vision could be implemented in the future on this basis.

### 5.2. PTT/PDT

PTT and PDT are two prominent approaches for tumor therapy. Their efficacy can be enhanced using NIR-II light, which has a significantly higher maximum permissible exposure for human tissue than visible and NIR-I light [21]. This property allows for the application of higher laser power densities. Song et al. developed an organic nanoparticle photosensitizer capable of both NIR-II fluorescence imaging and PDT. They constructed an endoscopic platform to achieve minimally invasive endoscopically guided interventional PDT, significantly inhibiting orthotopic pancreatic cancer and extending overall survival to 78 days in a mouse model [224]. Current NIR-II photosensitizers are often designed as theranostic agents that combine fluorescence imaging and photodynamic therapy functions, aiming to simplify the clinical workflow (Table 4). Nevertheless, the limitations of endoscopically guided interventional NIR-II PTT/PDT are similar to those of NIR-II fluorescence endoscopy [23,34]. As we referred, no novel tumor-targeted NIR-II probes or photosensitizers have entered clinical trials.

From a translational perspective, the clinical implementation of NIR-II endoscopic therapeutic strategies requires focused attention on endoscopy-specific feasibility and safety challenges. The narrow spaces and flexible anatomical constraints of MIS impose unique demands on NIR-II lasers and cameras—miniaturization is essential to ensure adequate flexibility during operation. Regarding NIR-II therapeutic agents, specifically photosensitizers and photothermal agents, they must exhibit stable photodynamic/photothermal activity and fully comply with existing regulatory requirements. Given that developing new agents is time-consuming and labor-intensive, a pragmatic approach could be to focus on repurposing or improving existing approved agents to meet the specific requirements of endoscopically guided interventional NIR-II PTT/PDT. Additionally, seamless integration into standard endoscopic workflows is critical to avoid disrupting clinical practice. Addressing these endoscopy-specific requirements is key to advancing NIR-II therapeutic technologies from preclinical research to clinical application.

## 6. The Future of Endoscopy: Integration with AI and 5G

The integration of AI and 5G into endoscopy represents a transformative frontier. AI-driven algorithms assist in lesion detection and characterization, and 5G enables real-time, high-resolution data transmission, facilitating telemedicine. This section explores the roles of AI and 5G in shaping the future of NIR-II endoscopic applications.

### 6.1. AI’s Transformative Role in Endoscopy

**Figure 6:**
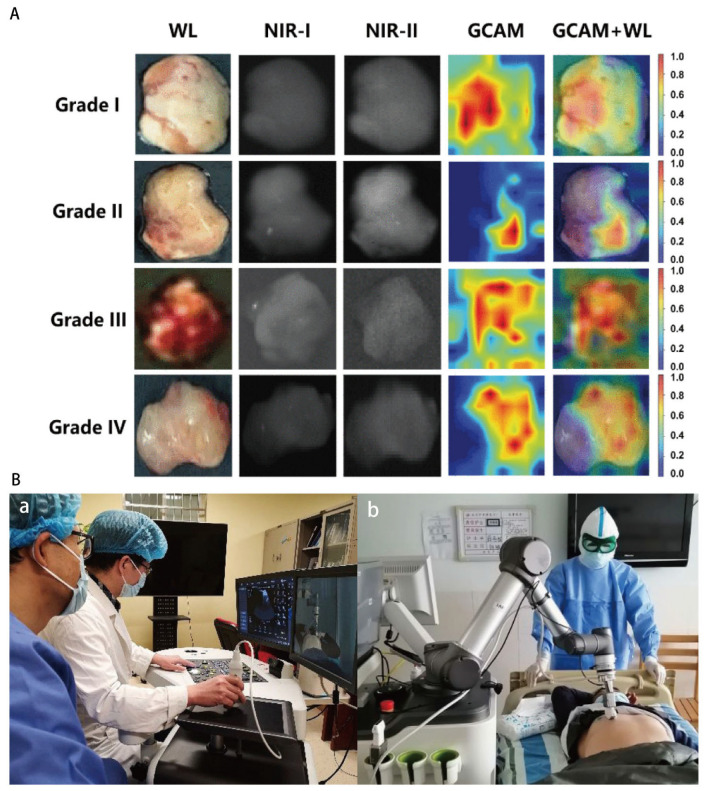
(**A**) The GCAM results of the NAS-based model on multi-modal intraoperative glioma images. GCAM: gradient-weighted class activation mapping and NAS: neural architecture search. Heatmaps visualize the attention of the NAS model, with red and dark blue indicating the most and least concerned regions. The result showed that the NAS model automatically found out the important and distinguishable parts on specimens that could hardly be recognized by human eyes. Reprinted from ref. [233], licensed under CC BY-NC-ND 4.0 (https://creativecommons.org/licenses/by-nc-nd/4.0/ accessed on 16 January 2026). Copyright © 2022 Jie Tian et al. (**B**) A robot-assisted tele-ultrasound examination. (**a**) The sonographer on the physician-side manipulates a simulated probe, and the control signal is captured through the ultrasound control panel and sent to the patient-side. (**b**) The robotic arm and a force sensor on the patient-side are used to execute the motion instructions from the physician-side and to complete the examination. Non-English text on the wall has no clinical relevance. Adapted with permission from ref. [234]. Copyright © 2020 Legao Chen et al.

AI has enabled data-driven algorithms capable of achieving human-level performance and beyond. Its applications include enhancing the quality of endoscopic imaging, optimizing intraoperative localization and guidance, and generating virtual H&E-stained images from heterogeneous input data (Figure 6A) [48,111,122,163,225,226,227,228]. Many AI endoscopy support systems, such as the GI Genius device (Medtronic Co., Minneapolis, MN, USA) and CAD EYE (Fujifilm, Tokyo, Japan), have received FDA clearance and been demonstrated as safe and effective tools for lesion detection, especially in early-stage colorectal lesions screening and surveillance [229,230]. Additionally, given the substantial advances in the field of IBD in recent years, the integration of AI into standard patient care is poised to expand significantly. AI’s utility is expected to encompass early risk evaluation, assistance in endoscopic image analysis, support for histopathological reporting, and the prediction of disease progression and treatment outcomes [231]. AI technology enhances detection rates, supports clinicians with limited experience, and automates image analysis, thereby alleviating workload and accelerating the clinical workflow [232].

As established previously, NIR-II endoscopy faces several critical translational barriers, including low signal-to-noise ratios under deep tissue imaging, constraints on real-time processing of large hyperspectral datasets, limited availability of high-repetition-rate pulsed lasers and scanning methods, a lack of clinically approved NIR-II fluorescent probes, and challenges in integrating the NIR-II into standard endoscopic workflows. In this context, AI is not merely a supplementary tool but a core enabler that directly addresses these bottlenecks. For instance, AI shows promise in mitigating the high cost of dedicated NIR-II equipment and improving compatibility with existing clinical workflows. Lu et al. proposed a novel solution for short-wave infrared (SWIR) imaging using a common silicon sensor and a three-stage image processing algorithm based on convolutional neural networks (CNNs). This approach may enable lower cost, higher resolution, better technical maturity and clinical compatibility relative to conventional InGaAs sensors [94]. Beyond addressing the practical hurdles outlined above, AI also demonstrates potential to uncover diagnostically relevant information from NIR-II endoscopic data. Olivo et al. developed an endoscopic probe capable of ultrabroadband hyperspectral sensing across wavelengths range from 420 nm to 1700 nm and captured datacubes from breast tissue samples to train deep learning models. Among the three models trained on paraffinized data, the NIR-II model achieved the highest accuracy, with validation and test accuracies of 99.66% and 99.70%, respectively. For deparaffinized samples, the NIR-II model outperformed models using visible and NIR-I spectral ranges, achieving validation and test accuracies of 99.90% and 99.92% [177]. For more studies on AI applications in NIR-II biomedical imaging, see Supplementary Table S1. Of course, the performance of AI models remains highly dependent on dataset characteristics, validation strategies, and eventual clinical deployment scenarios. These findings warrant further validation against appropriate clinical reference standards and rigorous study designs before clinical translation.

For medical applications like endoscopy, AI algorithms are trained on annotated tissue images to identify and classify pathological features. Model performance relies critically on dataset scale and annotation quality, highlighting the need for well-curated imaging databases tailored to NIR-II endoscopy [1]. Establishing dedicated, annotated imaging repositories is essential for advancing NIR-II endoscopy. Although clinical imaging data are growing rapidly, challenges such as data heterogeneity, multi-modality, and underrepresentation of rare or subtle lesions remain unresolved [235]. To improve reliability and translational potential, practical steps must be taken, including the development of multicenter-curated datasets, implementation of standardized annotation protocols, and integration of model calibration and uncertainty quantification into AI reporting [236,237,238]. Beyond strengthening regulations and standards, advancing explainable AI (XAI) systems that can transparently justify their predictions represents a key solution [239]. ENDOANGEL-ED (explainable diagnosis) provides predictions while revealing reasoning based on six feature indexes [240]. It offers valuable guidance for the development of an explainable AI-assisted NIR-II endoscopy system.

### 6.2. 5G Connectivity: Enabling Remote Endoscopy

There is a significant imbalance in the distribution of global medical resources [241]. Endoscopy, being highly operator-dependent, often requires skilled practitioners. Telemedicine offers a human-assisted approach to improve access to medical resources and popularize endoscopy (Figure 6B). Data transmission plays a pivotal role in the process. The advent of 5G has revolutionized data transmission, offering a theoretical peak downlink throughput of 10 Gbit/s per connection, reducing air link latency to below 1 ms (with end-to-end latency < 10 ms), and enabling connection density 100 times greater than 4G LTE [242,243]. Beyond connecting humans, 5G also supports connecting smart devices, including the 14.2 billion devices that constitute the Internet of Things (IoT) [244]. During the COVID-19 pandemic, 5G has supported various telemedicine applications, including robot-assisted tele-ultrasound, patient monitoring, computed tomography scans, and telesurgery [244,245,246,247,248,249].

To our knowledge, no research on NIR-II endoscopes for telemedicine has been conducted. But cases of tele-mentored laparoscopic surgeries exist. Niu et al. performed an ultra-remote radical cystectomy (network communication distance of nearly 3000 km) on a patient diagnosed with T2N0M0 stage bladder cancer using the “MicroHand” surgical robot. The 5G network was utilized throughout the procedure, with an average total delay of 254 ms [250]. Cai et al. reported an adverse event during an ultra-remote robot-assisted laparoscopic hepatobiliary and pancreatic surgery, where the ultrasonic knife failed to bite and cut tissue effectively. Although the issue was resolved by replacing the ultrasonic knife head, it disrupted the surgical workflow [251]. These results not only confirm that the system can deliver a highly accurate operational view and facilitate effective transfer of surgical skills, but also highlight the need for further improvements in the consumables of surgical robots.

Currently, 5G is mainstream for daily communication. For telemedicine applications, upgrading or buying specialized hardware and infrastructure incurs some costs, but this is not a major obstacle. With the development of communication technology, the next generation, 6G, will further advance telemedicine in the future.

## 7. Discussion and Outlook

### 7.1. Comparison with Other Publications

Several publications have highlighted recent advancements in NIR-II optical bioimaging. For example, Guo et al. discussed NIR-II imaging-guided drug delivery paradigms for the improvement of the prognosis of patients with tumors [252]. Wang et al. focused on NIR-II nanoprobes for biological imaging and examined the technical challenges for intravital NIR-II fluorescence imaging technology [23]. Our work focuses on integrated NIR-II endoscopic systems. We investigate their hardware–software co-design, real-time image processing, and clinical translation barriers across three key areas: endoscopic screening, diagnosis, and therapeutics. These challenges and corresponding solutions are systematically discussed in the corresponding sections. We not only evaluate the strengths and weaknesses of NIR-II endoscopy against clinical needs but also underscore its potential by integrating with AI and 5G technologies.

### 7.2. Current Challenges and Future Directions

Endoscopy has evolved from basic gastrointestinal screening to precision diagnosis and therapeutic assistance, expanding from visible and NIR-I to NIR-II wavelengths. While most NIR-II endoscopic explorations have been confined to preclinical and animal studies, their clinical application holds significant promise for the future.

For basic screening and precision diagnosis, a key challenge lies in addressing the interpretability of NIR-II images, particularly in establishing unified diagnostic criteria. Established standards, such as the pit pattern classification proposed by Kudo et al. for colorectal lesions, can serve as valuable references [253]. Kudo et al. analyzed the surface mucosal pits of lesions using a magnifying endoscope in vivo and established a correlation between pit patterns and the structure of the underlying crypts or glands, which has been adopted as a diagnostic reference for conventional colorectal endoscopy. NIR-II endoscopy could follow a similar strategy by establishing a correlation between NIR-II endoscopic images and different lesion types. This would significantly enhance image interpretability and facilitate its clinical translation. However, it is important to acknowledge that such criteria should be organ- and task-specific rather than relying on a single universal framework. Additionally, emerging imaging techniques should complement, rather than replace, conventional endoscopic methods. Combining various endoscopic modalities is essential for acquiring comprehensive biological information. Progress in various areas is gradually being used in advancing endoscopic technologies. For instance, researchers utilize 3D-printed optical components to optimize alignment and performance, and employ AI tools to reduce the cost of NIR devices [94,156]. These advances are critical for developing endoscope-compatible NIR-II systems that meet the physical constraints of clinical settings.

For surgical navigation and adjuvant therapy, advancements in manufacturing, assembly, and related fields can provide valuable insights, even if they are not specifically focused on the NIR-II region or endoscopy. The example of Cai et al. that we mentioned provides strong support for this point. Furthermore, while most current NIR-II PTT/PDT research remains confined to animal models, further large-animal studies are necessary to establish safety profiles, optimize dosage, and validate real-time imaging guidance under conditions that more closely mimic human anatomy.

For the medical application of AI and telecommunication, security issues are a critical consideration, encompassing both medical safety and data privacy. AI-based clinical assistance relies heavily on extensive training data, while telemedicine depends on 5G communication technology for real-time data transmission. Both approaches pose risks of patient data leakage and threats to medical safety. Therefore, robust data encryption and security protocols must be implemented to safeguard patient data privacy and ensure medical safety.

## 8. Conclusions

Expanding the spectrum to the NIR-II region offers new opportunities for endoscopy. While most NIR-II endoscopic explorations have been confined to animal studies, they hold significant promise for clinical applications. Imaging systems, fluorophores, detectors, and lasers all need to be optimized to enable better clinical NIR-II endoscopy. AI and 5G technologies provide a critical foundation for this and are paving the way for the future. It is anticipated that the next decade of NIR-II endoscopy will witness the "intelligent imaging systems", enabling more convenient, precise, effective, and intelligent clinical applications.

## Figures and Tables

**Table 1:** Dyes commonly used in clinic for chromoendoscopy.

Principle of Staining	Dyes	Origin of Contrast Improvement	Clinical Use	Advantages and Disadvantages
Absorptive	Lugol’s iodine [46,47,48]	Iodine stains glycogen in normal squamous epithelium dark brown or black; abnormal areas with less glycogen stain lightly or not at all.	Screening and detection of cervical neoplasia, cervical carcinoma, safe margins of oral and esophageal squamous cell carcinoma.	Staining contrast is clear and easy to assess, but the effect depends on tissue target substance levels. Some agents are contraindicated in specific patients.
	Methylene blue [49,50,51,52]	Methylene blue is rapidly absorbed by healthy intestinal mucosa but poorly taken up in inflamed or neoplastic areas.	Detection and diagnosis of intestinal metaplasia, sentinel lymph node biopsy, and esophageal fistula identification.	
	Toluidine blue [53,54,55,56,57]	Targeting acidic components like DNA, RNA, and proteoglycans, elevating mitotic activity and core-to-cytoplasmic ratio.	Detection of premalignant and malignant lesions of the oral cavity, oropharynx, esophagus and uterine cervix. Histological assessment of cartilaginous- and chondrogenic-differentiated tissues.	
Reactive	Acetic acid [46,58,59]	Acetic acid induces reversible protein denaturation in mucosa, yielding transient acetowhitening.	Detection of Barrett’s esophagus, esophageal adenocarcinoma and cervical intraepithelial neoplasia.	The staining process is rapid and reversible. But the staining is transient, necessitating prompt observation and judgment.
	Congo red [60]	Below a pH of 3, Congo red breaks down, shifting from red to black.	Identifying the stomach’s acid-secreting areas.	
Contrast	Indigo carmine [61,62,63]	It collects in crevices, accentuating tiny lesions and mucosal irregularities.	Distinguish neoplastic and nonneoplastic colonic polyps, highlight dysplastic lesion.	Only physical deposition; effective for superficial structural lesions but invalid for deep lesions.

**Table 2:** Commercially available virtual chromoendoscopy.

Imaging Processing Method	Modality	Specific Clinical Characteristics	Advantages and Disadvantages
Pre-processing	NBI [64]	Capillary network and submucosal vessels.	Real-time imaging via fast processing, but detection narrowly focused on vascular features.
	BLI [45,65]	Capillary network.	
	RDI [66,67]	Bleeding points and deep blood vessels.	
Post-processing	TXI [68]	Mucosal texture and subtle differences.	High contrast for mucosal details, yet with inherent processing delays.
	FICE [69]	Mucosal pit pattern and vascular.	
	i-scan [70]	Mucosal structure, vascular and depressed areas.	
	SPIES [71]	Mucosal pit pattern and vascular.	
Both pre- and post-processing	LCI [45]	Capillary network, texture and submucosal vessels.	Comprehensive characterization of vascular, texture, and submucosal features comes with complex imaging algorithms and higher hardware requirements.
	i-scan OE [72]	Capillary network, texture and submucosal vessels.	

Pre-processing methods enhance contrast by modifying the illumination spectrum, and post-processing methods do so through digital image processing.

**Table 3:** Performance comparison of endoscopic imaging modalities.

Endoscopic Imaging Technology	Resolution	Molecular Contrast	Imaging Depth	Main Limitation
Confocal laser endoscopy [102,115,153]	Axial: ~5–10 µm Lateral: ~0.5–5 µm	N	~40–200 µm	Insufficient penetration depth Limited field of view
Endocytoscopy [102]	Lateral: ~1–5 µm	N	~5–50 µm	Insufficient penetration depth Limited field of view
Hyperspectral endoscopy [120,123]	Spectral resolution: 1–10 nm	Y	Millimeter-scale	Long imaging time Low spatial resolution
Endoscopic optical coherence tomography [138,141,155,158,159]	Axial: ~5–15 µm Lateral: ~5–30 µm	N	1–3 mm	Challenges in miniaturization High equipment costs
Multiphoton endoscopy [106,107,108,109,110,160,161]	Axial: ~0.5–3 µm Lateral: ~5–15 µm	Y	100–300 µm	Complex and expensive system Risk of phototoxicity
Photoacoustic endoscopy [101,162,163,164]	Axial: ~1.5–150 µm Lateral: <200 µm	Y	~1 mm–4 cm	Complex and expensive system Balance imaging depth and resolution
Ultrasonic endoscopy [165,166]	Axial: ~50–800 mm Lateral: <300 µm	Y	2–8 cm	Limited field of view Balance imaging depth and resolution

## Data Availability

No new data were created or analyzed in this study. Data sharing is not applicable to this article.

## Supplementary Materials

The following supporting information can be downloaded at: https://www.mdpi.com/article/10.3390/diagnostics16070986/s1, Supplementary Table S1. Summary of main AI applications in NIR-II biomedical imaging since 2020 [254,255,256,257,258,259,260,261,262,263,264,265,266,267,268,269,270].

## Abbreviations

The following abbreviations are used in this manuscript:

GI	Gastrointestinal
WLE	White-light endoscopy
CLE	Confocal laser endomicroscopy
EC	Endocytoscopy
HSE	Hyperspectral endoscopy
MPE	Multiphoton endoscopy
OCT	Optical coherence tomography
PAE	Photoacoustic endoscopy
NIR	Near-infrared
NIR-I	The first near-infrared window
SNR	Signal-to-noise ratio
NIR-II	The second near-infrared window
AI	Artificial intelligence
5G	Fifth-generation mobile communication technology
FOV	Field of view
VCE	Virtual chromoendoscopy
SD	Standard definition
HD	High definition
IBD	Inflammatory bowel disease
MTF	Modulation transfer function
InGaAs	Indium gallium arsenide
nA	Nanoampere
2D	Two-dimensional
RDI	Red dichromatic imaging
NBI	Narrow-band imaging
FDA	Food and Drug Administration
H&E	Hematoxylin and eosin
NIR/ICG	Near-infrared imaging with indocyanine green
PTT/PDT	Photothermal/photodynamic therapy
FWHM	Full-width at half-maximum
3D	Three-dimensional
3PEF	Three-photon excitation fluorescence
THG	Third harmonic generation
CARS	Coherent anti-Stokes Raman scattering
TPF	Two-photon fluorescence
SHG	Second harmonic generation
PAI	Photoacoustic imaging
US	Ultrasound
EMA	European Medicines Agency
MIS	Minimally invasive surgery
AIE	Aggregation-induced emission
DOF	Degree-of-freedom
SWIR	Short-wave infrared
CNNs	Convolutional neural networks
LTE	Long Term Evolution
IoT	Internet of Things
