# Supplementary material for: Advances and Opportunities in NIR-II Endoscopy: From Diagnosis to Therapeutic Applications
Source: Diagnostics (Basel). 2026 Mar 25;16(7):986. doi: 10.3390/diagnostics16070986 (PMC13072825; doi:10.3390/diagnostics16070986)
Supplement: Supplementary file 1 [file diagnostics-16-00986-s001.zip › diagnostics-4176849-supplementary.pdf]

Supplementary Information

Advances and Opportunities in NIR-II Endoscopy: From Diagnosis to Therapeutic Applications

Jing Luo [1], Xiaofan Du [1], Sijia Wang [1], Cuiping Yao [1] and Jing Wang [1,*]

[1] Institute of Biomedical Photonics and Sensing, Key Laboratory of Biomedical Information Engineering of Ministry of Education, School of Life Sciences and Technology, Xi'an Jiaotong University, Xi'an 710049, China; 1474642445@stu.xjtu.edu.cn (J. L.); dxf03@xjtu.edu.cn (X. D.); wang_sijia@xjtu.edu.cn (S. W.); zsycp@mail.xjtu.edu.cn (C. Y.); wangjing@xjtu.edu.cn (J. W.)

* Correspondence: wangjing@xjtu.edu.cn

Contents

Supplementary Table S1: Summary of main AI applications in NIR-II biomedical imaging since 2020.

Supplementary Table S1. Summary of main AI applications in NIR-II biomedical imaging since 2020.

Ref.	Field	Study feature	Main finding
Zhenhua Hu et al.[1]	NIR-II FMT	Proposed a diffusion-based data augmentation strategy with NIR-II-specific optical guidance to generate diverse synthetic training samples.	Augmentation reduced FID by 56.7% and improved IS by 21.5%, enhancing FMT reconstruction accuracy for tumors in simulation and in vivo.
Dayong Jin et al.[2]	NIR-II fluorescence imaging with light-sheet illumination	Combined light-sheet illumination, time-gated detection, and deep learning for high-contrast, high-resolution 3D volumetric imaging.	Achieved 220 μm axial resolution and 0.45 contrast, resolved 100 μm vessels from 0.07 contrast regions; enabled rapid 3D imaging of vascular networks >1 mm deep within 6 min.
Lining Sun et al.[3]	NIR-II fluorescence imaging with lanthanide-doped nanocrystals	Designed excitation-dependent nanocrystals with switchable visible/NIR-II emission, combined with deep learning to integrate narrow visible emission and deep NIR-II penetration.	Achieved high SNR and narrow emission peak through phantom tissue, showing potential for intelligent optical materials and in vivo information security applications.
Fuchun Chen et al.[4]	NIR-II fluorescence vascular imaging	Proposed an unsupervised attention-GAN with attention loss to translate vascular masks into realistic NIR-II fluorescence vascular images, no paired training data needed.	Outperformed 8 baselines in visual and quantitative metrics, generating high-fidelity synthetic data to solve limited dataset issues in NIR-II imaging.
Ye Tian et al.[5]	NIR-IIa to NIR-IIb image translation	Developed BRCycle-GAN, a CNN trained on only 63 images, to upgrade NIR-IIa images to NIR-IIb quality.	Outperformed prior models in PSNR and cosine similarity, enabling high-quality NIR-IIb imaging without toxic probes and limited training data.
Jun Qian et al.[6]	NIR-II fluorescence volumetric microscopy	Combined ETL with two deep learning networks (SRN for enhancement, CVI for axial interpolation) for rapid 3D deep-tissue vascular imaging.	Achieved 4.2 fps volumetric imaging over 200 μm depth in mouse brain; 16-fold axial upsampling enabled smooth 3D reconstruction and quantitative blood velocity measurement at varied depths.
Jun Qian et al.[7]	NIR-II fluorescence image resolution enhancement	Fine-tuned Real-ESRGAN to overcome detector resolution limits in NIR-II imaging, validated in whole-body vascular, NIR-IIc, and diabetic foot clinical scenarios.	Outperformed bilinear and bicubic interpolation in PIQE and FWHM; generalized to untrained NIR-IIc data (abdomen, intestine, leg), enabling clearer vascular visualization for clinical surgical guidance.
Ye Tian et al.[8]	NIR-II imaging-guided cancer	Designed zwitterionic polymer conjugate for Atezolizumab delivery	Conjugate enhanced tumor accumulation and T cell proliferation,

	immunotherapy	with tumor-targeted release and T cell redox homeostasis restoration; visualized in vivo dynamics via deep learning-enhanced NIR-II imaging.	suppressed colorectal tumor growth/recurrence by alleviating T cell ferroptosis, and prolonged survival in murine models.
Xuechuan Hong et al.[9]	NIR-II imaging-guided siRNA therapy for ischemic stroke	Developed long-circulating YWFC NPs crossing blood-brain barrier for NLRP3 siRNA delivery; integrated deep learning to optimize NIR-II imaging of cerebral infarct penumbra.	Image-guided siNLRP3 delivery enhanced BBB penetration and yielded significant therapy in MCAO mice; deep learning-improved NIR-II imaging achieved superior SNR at 72 h post-stroke.
Zhenhua Hu et al.[10]	NIR-II fluorescence vascular image segmentation	Proposed CALFNet: a context-aware local feature network with UNet-like structure, ResNet encoder, Mamba-based global context module, and feature-enhancement module for low-contrast vessel segmentation.	Outperformed competing methods on NIR-II vascular and retinal datasets, enabling more accurate, robust fine vessel segmentation in low-contrast regions and boosting clinical utility of NIR-II imaging.
Ruichan Lv et al.[11]	NIR-II imaging-guided magnetic nanorobots for precision biomedicine	Developed magnetic nanorobot platform with Fe_2O_3 and rare-earth nanoparticles for NIR-II/MRI/PA multimodal imaging and antibody-magnetic dual targeting; integrated deep learning for NIR-II enhancement and temperature sensing.	Achieved rapid tumor targeting (<5 s) and precise localization; real-time temperature detection (24.6–41.5 °C, R^2=0.973, RMSE=1.50 °C); deep learning enabled simultaneous surgical boundary definition and temperature visualization.
Xiaogang Qu et al.[12]	NIR-II-responsive HOF-based NSC encapsulation for Alzheimer's disease therapy	Designed HOF encapsulated NSCs doped with PCNs; PCNs exhibited catalase/SOD-like activities for oxidative stress resistance, while NIR-II triggered thermal-responsive HOF dissociation for on-demand NSC release.	Encapsulation preserved NSC stemness, reduced transplantation damage and apoptosis; NIR-II-triggered release enhanced cell viability, promoted neurogenesis, and ameliorated cognitive impairment in AD mice.
Lining Sun et al.[13]	Multimodal emission nanocrystals for NIR-II information security	Developed Er^{3+}-based lanthanide nanocrystals with switchable visible/NIR-II emission ratios under 980/808/1535 nm excitation, combined with deep learning for information decoding.	Realized switchable output via varied excitation wavelengths; demonstrated tissue-penetrating visible information storage and decoding using multimodal emissions and deep learning.
Hong bo et al.[14]	NIR-II FMT reconstruction	Proposed DSPGN, a deep system prior graph convolution network with spatial/topological information and attention mechanism for improved FMT morphological reconstruction.	Outperformed existing methods in localization accuracy and shape recovery in simulation and in vivo, showing great potential for NIR-II FMT applications.

Bhargava Rohit et al.[15]	Infrared (IR) spectroscopic imaging	Developed a deep learning framework with IR-SEG (CNN for spatial-spectral segmentation) and IR-REC (GAN for full dataset reconstruction from incomplete spatial-spectral data).	IR-SEG achieved higher accuracy with ~50% fewer features; IR-REC enabled up to 20-fold faster acquisition with minimal loss; single-band IR imaging surpassed diffraction limit in spatial detail.
Jie Tian et al.[16]	NIR-II/fluorescence image denoising	The proposed DEQ-UMamba combines proximal gradient descent and spatial-frequency feature learning to decouple complex noise, utilizing deep equilibrium learning for stable convergence with fewer parameters.	Outperformed state-of-the-art methods on clinical and in vivo datasets with fewer parameters, enabling interpretable noise suppression and high-quality, cost-effective clinical molecular imaging.
Gao Feng et al.[17]	fNIRS-DOT neuroimaging	Developed a model-informed deep learning method combining semi-3D DOT (S3D-DOT) and DCNN to reduce ill-posedness, suppress cardiac/respiratory and random noise, and improve reconstruction accuracy.	Achieved mean SSIM >0.998 in simulations, outperforming conventional ART in accuracy and speed; robust under strong noise and dual-target conditions; validated in phantoms; 3D-DCNN extension enabled accurate time-resolved absorption reconstruction.

FMT: Fluorescence molecular tomography; FID: Fréchet Inception Distance; IS: Inception Score; SNR: Signal-to-noise ratio; GAN: Generative adversarial network; CNN: Convolutional neural network; PSNR: Peak Signal -to-noise ratio; ETL: Electrically tunable lens; NIR-IIa, NIR-IIb, NIR-IIc: Subwindows of the NIR-II region (1,000–1,300 nm, 1,500–1,700 nm, 1700-1880 nm, respectively); PIQE: Perception-based image quality evaluator; FWHM: Full width at half maximum; siRNA: Small interfering RNA; BBB: Blood-brain barrier; MCAO: Middle cerebral artery occlusion; HOF: Hydrogen-bonded organic framework; NSC: Neural stem cell; PCN: Porous carbon nanosphere; SOD: Superoxide dismutase; AD: Alzheimer's disease; DOT: Diffuse optical tomography; DCNN: Deep convolutional encoder-decoder; SSIM: Structure similarity index; ART: Algebraic reconstruction technique. Unless otherwise noted, all works listed above are yet to be clinically validated

References

1. Huang, Q.; Li, C.; Xiao, A.; Tian, J.; Hu, Z. DANG: Data Augmentation Based on NIR-II Guided Diffusion Model for Fluorescence Molecular Tomography. *IEEE Trans. Comput. Imaging* **2026**, *12*, 128–141, doi:10.1109/TCI.2025.3643313.

2. Wu, S.; Yang, Z.; Ma, C.; Zhang, X.; Mi, C.; Zhou, J.; Guo, Z.; Jin, D. Deep Learning Enhanced NIR-II Volumetric Imaging of Whole Mice Vasculature. *Opto-Electron. Adv.* **2023**, *6*, 220105, doi:10.29026/oea.2023.220105.

3. Song, Y.; Lu, M.; Xie, Y.; Sun, G.; Chen, J.; Zhang, H.; Liu, X.; Zhang, F.; Sun, L. Deep Learning Fluorescence Imaging of Visible to NIR-II Based on Modulated Multimode Emissions Lanthanide Nanocrystals. *Adv. Funct. Mater.* **2022**, *32*, 2206802, doi:10.1002/adfm.202206802.

4. Fang, L.; Sheng, H.; Li, H.; Li, S.; Feng, S.; Chen, M.; Li, Y.; Chen, J.; Chen, F. Unsupervised Translation of Vascular Masks to NIR-II Fluorescence Images Using Attention-Guided Generative Adversarial Networks. *Sci Rep* **2025**, *15*, 6725, doi:10.1038/s41598-025-91416-y.

5. Wang, Y.; Wang, L.; Tian, Y. BRCycle-GAN: A Near-Infrared Fluorescence Image Processing Network Based on a Small Training Set. *IEEE Access* **2024**, *12*, 94520–94526, doi:10.1109/ACCESS.2024.3421525.

6. Peng, S.; Zhang, Y.; Mou, X.; Wu, T.; Zhang, M.; Qian, J. Deep Learning-Enhanced NIR-II Fluorescence Volumetric Microscopy for Dynamic 3D Vascular Imaging. *J. Innov. Opt. Health Sci.* **2025**, *18*, 2550013, doi:10.1142/S1793545825500130.

7. Peng, S.; Zhang, Y.; Liu, X.; Fan, X.; Lin, H.; Qian, J. Deep Learning-Based Resolution Enhancement Method for NIR-II Fluorescence Imaging (Invited). *Laser Optoelectron. Prog.* **2025**, *62*, 1817022, doi:10.3788/LOP251220.

8. Guo, R.; Chen, R.; Rao, Z.; Wang, L.; Xi, J.; Zhang, Y.; Guo, W.; Tian, Y. Boosting Checkpoint Blockade Immunotherapy with T Cell Membrane Redox Homeostasis Regulation and Deep Learning Enhanced NIR-II Imaging. *Adv. Healthc. Mater.* **2025**, *14*, 2500769, doi:10.1002/adhm.202500769.

9. Yu, K.; Fu, L.; Chao, Y.; Zeng, X.; Zhang, Y.; Chen, Y.; Gao, J.; Lu, B.; Zhu, H.; Gu, L.; et al. Deep Learning Enhanced Near Infrared-II Imaging and Image-Guided Small Interfering Ribonucleic Acid Therapy of Ischemic Stroke. *ACS Nano* **2025**, *19*, 10323–10336, doi:10.1021/acsnano.4c18035.

10. Han, K.; Xiao, A.; Tian, J.; Hu, Z. Mamba-Based Context-Aware Local Feature Network for Vessel Detail Enhancement. *Comput. Med. Imaging Graph.* **2025**, *125*, 102645, doi:10.1016/j.compmedimag.2025.102645.

11. Li, W.; Lin, B.; Li, B.; Zhang, P.; Ju, Z.; Ansari, A.A.; Lv, R. Deep Learning Enabled Magnetic/Rare Earth Hybrid Nanorobots for Multi-Modal Bioimaging and Temperature Sensing with Surgical Boundary Determination. *Sens. Actuator B-Chem.* **2026**, *455*, 139673, doi:10.1016/j.snb.2026.139673.

12. Yu, D.; Zhang, H.; Liu, Z.; Liu, C.; Du, X.; Ren, J.; Qu, X. Hydrogen-Bonded Organic Framework (HOF)-Based Single-Neural Stem Cell Encapsulation and Transplantation to Remodel Impaired Neural Networks. *Angew. Chem.-Int. Edit.* **2022**, *61*, e202201485, doi:10.1002/anie.202201485.

13. Song, Y.; Lu, M.; Mandl, G.A.; Xie, Y.; Sun, G.; Chen, J.; Liu, X.; Capobianco, J.A.; Sun, L. Energy Migration Control of Multimodal Emissions in an Er3+-Doped Nanostructure for Information Encryption and Deep-Learning Decoding. *Angew. Chem.-Int. Edit.* **2021**, *60*, 23790–23796, doi:10.1002/anie.202109532.

14. Wang, B.; Li, S.; Zhang, H.; Li, J.; Zhang, L.; Yu, J.; He, X.; Guo, H. Deep System Prior Based Graph Convolution Network for NIR-II Fluorescence Molecular Tomography. *Comput. Meth. Programs Biomed.* **2025**, *270*, 108948, doi:10.1016/j.cmpb.2025.108948.

15. Falahkheirkhah, K.; Yeh, K.; Mittal, S.; Pfister, L.; Bhargava, R. Deep Learning-Based Protocols to Enhance Infrared Imaging Systems. *Chemometrics Intell. Lab. Syst.* **2021**, *217*, 104390, doi:10.1016/j.chemolab.2021.104390.

16. Fu, L.; Li, L.; Lu, B.; Guo, X.; Shi, X.; Tian, J.; Hu, Z. Deep Equilibrium Unfolding Learning for Noise Estimation and Removal in Optical Molecular Imaging. *Comput. Med. Imaging Graph.* **2025**, *120*, 102492, doi:10.1016/j.compmedimag.2025.102492.

17. Li, T.; Liu, D.; Zhang, P.; Li, Z.; Gao, F. Deep Convolutional Encoder Decoder Neural Network Approach for Functional Near Infrared Spectroscopic Imaging. *Chin. J. Lasers* **2023**, *50*, 2107107, doi:10.3788/CJL230734.
